# Pubic Hair Shaving Is Correlated to Vulvar Dysplasia and Inflammation: A Case-Control Study

**DOI:** 10.1155/2017/9350307

**Published:** 2017-08-27

**Authors:** Meike Schild-Suhren, Amr A. Soliman, Eduard Malik

**Affiliations:** Universitätsklinik für Gynäkologie und Geburtshilfe, Klinikum Oldenburg AöR, Fakultät für Medizin und Gesundheitswissenschaften, Carl von Ossietzky Universität Oldenburg, Oldenburg, Germany

## Abstract

**Objective:**

The risk factors for vulvar dysplasia and infections are not fully known. In this study, we aimed to investigate the correlation between pubic hair shaving and the occurrence of vulvar inflammation, dysplasia, and cancer.

**Methods:**

This study was performed between January 2013 and December 2016 in which a standardized questionnaire concerning genital hair shaving was administered to vulvar dysplasia and cancer patients and healthy participants. The presence of human papilloma virus (HPV) infection and the occurrence of genital inflammation were documented.

**Results:**

We recruited 49 patients with vulvar dysplasia or cancer and 234 healthy women as a control group. Smoking, HPV infection, genital inflammation, and complete pubic hair removal were significantly more common in the vulvar dysplasia/cancer group. Pubic hair shaving per se presented a clear association with vulvar dysplasia/cancer. Shaving the labia majora in particular showed also an association.

**Conclusion:**

Our findings suggest that partial or complete pubic hair shaving using a razor is correlated with and could be a potential risk factor for the development of genital inflammation, vulvar dysplasia, and malignancies. These results need to be confirmed in larger studies. HPV status and genital inflammation should be documented by medical personnel.

## 1. Introduction

The incidence of vulvar intraepithelial neoplasia (VIN) has increased 5-fold since the 1970s, and the disease now affects 7/100,000 women, making it the most common preinvasive disease of the vulva in Germany. The incidence of vulvar cancer has risen by approximately 20% since the 1970s, and the disease now affects 12/100,000 women in Germany [1]. The risk factors for vulvar dysplasia and invasive cancer are human papilloma virus (HPV) infection, cervical cancer, nicotine consumption, condylomas, genital herpes, human immunodeficiency virus infection, and immunosuppression [2].

VIN was classified by the international society for the study of vulvovaginal disease (ISSVD) in 2004 into the following categories [3, 4]:

- Classical VIN: including lesions formerly known as VIN II and VIN III, bowenoid, condylomatous, and mixed form
- Differentiated VIN: previously known as VIN of simple type.

Classical VIN is the most common form of vulvar dysplasia with an incidence of 5/100.000 women [5, 6]. The common age of patients at presentation is between 30 and 40 years. The progression rate of classical VIN to invasive cancer is 3.3–5.7% within 2.4 to 13.8 years [7, 8]. Differentiated VIN constitutes only 5–10% of all vulvar preinvasive lesions and is often found associated with lichen sclerosis [5]. In 33% of cases, it progresses to carcinoma [5].

VIN presents clinically as elevated or flat vulvar lesions with a color that varies from white to grey or red to brown or black [9]. Vulvar cancer appears as a vulvar lump or mass that might also be ulcerated [10].

The aforementioned factors cannot be solely responsible for the dramatic increase in the incidence of the disease. There should be other factors playing a role in the development of the disease.

Pubic hair removal may be a risk factor for VIN or correlated with the increasing incidence of VIN. Until the 1980s, it was considered normal for women to shave only the bikini zone and armpits, while the latest trend, especially among younger women, is total pubic hair removal [11]. This trend can be observed in magazines like Playboy, which demonstrate a transition from completely hairy pubic areas to different degrees of shaving and, finally, to total pubic hair removal [12].

While female body hair removal is generally practiced according to psychosocial reasons regardless of the body part the hair being removed from, genital hair removal does have health risks because pubic hair has a definite biological purpose as a safety net to protect the vulva from bacterial infections [13]. A study based on data from the “National Electronic Injury Surveillance System” showed that the number of emergency room consultations due to genital injuries after shaving increased 5-fold from 2002 to 2010. The authors stated that 81.9% of the injuries were caused by nonelectric shaving methods. The long-term consequences of these injuries are not yet clear [14]. Another study gathered data from 1110 college students in the United States, who reported on the complications they experience during genital shaving, namely, pain, rash, cuts, and itching with genital itching as the most common complication of genital hair removal being reported by 80.2% of the participants in this study [15]. The Federal Center of Health Education in Germany, with the University of Hamburg, interviewed 160 teenagers aged 16–19 years and found that 94% of the female and 81% of the male participants shaved their pubic hair [16]. A study at the University of Leipzig in 2009 investigated the shaving patterns of 2,512 young participants aged 18–25. In this study, 88% of female and 67% of male participants admitted to practicing partial or total pubic hair shaving. This trend strongly declined at the beginning of the 31st year of age [17].

The aim of this study was to investigate the correlation between pubic hair shaving and vulvar dysplasia and cancer.

## 2. Methods

This was a case-control study involving patient interviews between January 2013 and December 2016. All patients were aged 20–70 years, visited the University Clinic of Obstetrics and Gynecology at Oldenburg Hospital between January 1, 2013, and June 30, 2015, and were diagnosed with and treated for VIN or vulvar cancer.

The control group consisted of healthy women aged 20–70 years who attended an outpatient gynecology clinic in Zetel (Lower Saxony) for regular cancer screening between October 1, 2014, and December 31, 2014. The exclusion criteria for the control group were as follows: current cancer or previous treatment for cancer. The women filled out a questionnaire while the gynecologist was taking their medical histories. Both controls and patients received and completed identical consent forms and questionnaires.

The questionnaire (enclosed) covered the following parameters: age, smoking, HPV infection, genital infections, patterns of shaving, degree of hair removal (the areas shaved), and the duration that the patient/healthy participant had been practicing shaving (in years). HPV infection and inflammation were documented according to self-assessments performed by each participant.

The participants marked the areas of hair removal and the patterns were divided into the following four categories: total shaving, shaving (including the labia majora), shaving (excluding the labia majora), and no shaving. Participants were presented with a diagram as a supplement to the questionnaire to facilitate this process.

Forty-nine of 51 individuals were immediately ready to participate in the study and answered the questionnaire. All healthy patients addressed in the gynecology practice were ready to take part.

Baseline characteristics, recorded as means with standard deviations and medians with 1st and 3rd quartiles, were included in the statistical analysis. The distribution of characteristics was described using absolute and relative frequencies. The relationships between two characteristics were illustrated using contingency tables and tested using the chi-square test. Fisher's exact test was used instead of the chi-square test in cases of small sample sizes. Logistic regression analysis was performed on dichotomous characteristics to exclude interchangeable effects. Test results with *p* values < 0.05 were considered statistically significant (i.e., an alpha level of 0.05 was used for all statistical tests). Since this was an exploratory study, the statistical results are descriptive rather than confirmatory. The statistical analyses were performed using R (version 3.0.1) (https://www.r-project.org) and Statistical Analysis System (SAS) version 9.4 (2013) (SAS institute, Cary, NC, USA).

### 2.1. Role of the Funding Source

This study was supported by internal funding sources of the University Women's Hospital, Klinikum Oldenburg AöR, Carl von Ossietzky Universität Oldenburg, Oldenburg, Germany.

## 3. Results

The sample population included 49 patients with VIN I, VIN II/III, or vulvar cancer and 234 healthy women (control group). The characteristics of the cohort are presented in Table 1.

The effect of pubic hair shaving on the development of VIN III (or, rather, vulvar cancer) was investigated by logistic regression analysis adjusted for age and smoking status. The results are shown in Table 2.

The influence of the extent of hair removal on the incidence of vulvar dysplasia or cancer was determined using logistic regression analysis adjusted for age and smoking. The results are shown in Table 3.

## 4. Discussion

Female body hair and in particular pubic hair removal are sociopsychologically driven practices that are based on the perception that the female body is not accepted as such and must be altered in order to be perceived as attractive and acceptable [18]. The medical and health consequences of this practice are however not extensively enough studied. As much as 60% of the participants in a questionnaire regarding pubic hair removal experienced at least one health complication because of the hair removal, of which the most common were epidermal abrasion and ingrown hairs. Only 4% of the participants had seen a healthcare provider for a complication related to hair removal and only 4% discussed safe removal practices with their doctor [19]. This reflects the magnitude of the health risks related to this practice and the lack of knowledge about how to practice it safely.

In this study, we investigated the genital shaving patterns of healthy women and women with VIN or vulvar cancer who were aged 20–70 years. We found that age, the extent of genital shaving, and the frequency of self-diagnosed inflammation in the genital area were correlated with VIN or vulvar cancer. These results indicate that pubic hair shaving may be associated with vulvar dysplasia and cancer.

In this cohort, the younger women shaved their pubic area significantly more often than older women, and the extent of shaving decreased with advancing age. We noticed that the women with vulvar dysplasia or cancer reported that they completely or partially shave their pubic hair more often and experienced recurrent inflammation. These results are in accordance with those of a previous study [17], which showed that shaving the pubic hair leads to a 4-fold increase in the risk of vulvar dysplasia or cancer. Our analysis also demonstrated a correlation to the extent of shaving. Complete shaving and shaving only the labia majora were correlated to the occurrence of dysplasia and cancer more frequently than those who did not perform this extent of shaving. The correlation between age and the degree of shaving in our study was similar to that reported by a previous study [17].

Women who completely shave their pubic hair or shave the labia majora show more correlation with developing vulvar dysplasia and cancer. A possible explanation for this may be that shaving the genitals leads to an increased risk of inflammation. This would also account for the higher rates of VIN and cancer in the anterior commissure [20]. Other means of removing pubic hair, such as waxing, were not accessible to the participants in the rural area in northwestern Germany where the study was conducted. Therefore, this study could only analyze the impact of shaving and not other forms of hair removal.

This study has several limitations. First, it was a single-center study and included a relatively small number of patients. Second, inflammation in the pubic area and HPV status were self-reported by patients and not medically confirmed, which is a source of bias and may have led to an unreliable incidence rate. Third, HPV-induced disease and non-HPV-induced disease were not compared in this study. A possible theoretical explanation for a confounding relationship between shaving and HPV infection is that the manipulation of skin by a razor may facilitate the penetration of the virus into the skin cells. Another point that might have affected the validity of the results of our survey is the extent of pubic hair removal over the years in which this was practiced. We surveyed our participants for the length of years they performed pubic hair removal but did not gather data about the duration of years for which each of the patterns of pubic hair removal was performed. This might also be a relevant issue that affects our results. It should be considered in further studies. The age of the participants was significantly higher in the cases group and was not matched to the age of the participants in the control group. This is attributed to the fact that vulvar cancer affects primarily older women. This can also affect the validity of the results.

In conclusion, the present study is the first of its kind in Germany to investigate shaving patterns in younger and older women and to identify a possible correlation between pubic hair shaving and vulvar dysplasia and cancer. Partial or complete pubic hair shaving using a razor is correlated with and could be a potential risk factor for genital inflammation, vulvar dysplasia, and malignancies. These results need to be confirmed and explored in future studies including larger numbers of patients. Furthermore, HPV status and genital inflammation should be documented by medical personnel in clinical practice.

## Supplementary Material

The questionnaire form used to acquire data for this study (translated to English from German).

## Figures and Tables

**Table 1:** Descriptive representation of cases and controls.

	Cases (*n* = 49)		Control (*n* = 234)		*p* value
	Number	Percentage	Number	Percentage	
*Age* ^**∗**^ * (years)*	51.1 (10.8)		43.2 (13.9)		<0.001
*Type of cancer/VIN*					
VIN I	9	18			
VIN II/III	25	51			
Vulvar cancer	15	31			
*Genital hair removal*					
Yes	39	80	160	68.4	0.1
Not at all	10	20	74	31.6	
*Extent of hair removal*					
Not at all	10	18	74	31.6	0.02
Without labia majus	6	14	59	25.2	
With labia majus	4	8	10	4.3	
Complete genital hair removal	29	59	91	38.9	
*Method of hair removal*					
Not at all	10	20	74	31.6	0.02
Shaving	36	73	158	67.5	
Waxing	3	6	1	0.4	
Other	0	0	1	0.4	
*For how many years the participant removed the pubic hair* ^**∗**^	10 (5–20)		10 (0–14)		0.05
*Inflammation*					
Yes	40	81,63	62	26.5	<0.0001
No	9	18,37	172	73.5	
*HPV status*					
Positive	21	42,86	29	12.4	<0.0001
Negative	21	42,86	194	82.9	
Not known	7	14,29	11	4.7	
*Smoking status*					
Yes	27	55,10	69	29.5	<0.001
No	11	22,45	114	48.7	
History	11	22,45	51	21.8	

^**∗**^Mean (standard deviation); categorical variables: Chi-square test or Fisher's exact test; metric variables: ANOVA.

**Table 2:** Logistic regression for the development of vulva dysplasia/VIN according to hair removal adjusted for smoking status and age.

	Coefficient of regression	Standard error	*p* value	Odds ratio	95% confidence interval (CI)
Genital hair removal	1.40	0.46	0.003	4.04	1.63–10.01
Age	0.08	0.02	<0.0001	1.08	1.05–1.12
Smoking status	1.41	0.43	0.001	4.10	1.79–9.44
Smoking history	0.70	0.48	0.1	2.01	0.78–5.16

**Table 3:** Logistic regression of the occurrence of vulva dysplasia/VIN by the extent of hair removal, adjusted for age and smoking status.

	Coefficient of regression	Standard error	*p* value	Odds ratio	95% confidence interval
Without shaving labia majora	0.32	0.60	0.6	1.38	0.43–4.42
Shaving labia majora	2.7	0.89	0.002	15.17	2.68–85.94
Complete	2.64	0.59	<0.0001	14.07	4.44–44.63
Age	0.11	0.019	<0.0001	1.12	1.08–1.16
Smoking status	1.28	0.46	0.005	3.61	1.48–8.80
Smoking history	0.87	0.51	0.09	2.38	0.89–6.40

## Abbreviations

HPV: Human papilloma virus
VIN: Vulvar intraepithelial neoplasia.
