# Supplementary material for: Pubic Hair Shaving Is Correlated to Vulvar Dysplasia and Inflammation: A Case-Control Study
Source: Infect Dis Obstet Gynecol. 2017 Aug 27;2017:9350307. doi: 10.1155/2017/9350307 (PMC5591962; doi:10.1155/2017/9350307)

Dear Ms …

We are conducting a study to determine the possible correlation between shaving the pubic hair and malignancy of labia. We could really use your help. We have put together a few questions regarding the same, and would like to know your answers. We understand the sensitive nature of these questions and assure you that your name and responses will remain entirely anonymous. Thank you very much in advance for your participation.

How old are you? ______

Do you smoke? Yes O No O

Did you smoke in the past? Yes O No O

Do you have a HPV infection? Yes O No O I don’t know O

Do you or have you had inflammation in the pubic area? Yes O No O

Do you regularly shave or wax your pubic region? Yes O No O

If yes how? Shave O Wax O

How long has it been since you’ve been practicing it? ______ Years?

Which areas? All O

If not all, then please tick or plotting which areas are shaved


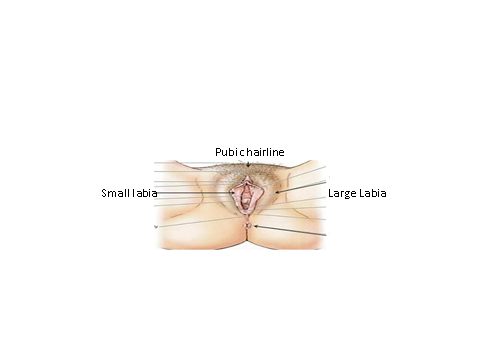

Supplement: Supplementary file 1 — The questionnaire form used to acquire data for this study (translated to English from German). [file 9350307.f1.docx]
